# Factors Predicting Favorable Short-Term Response to Transforaminal Epidural Steroid Injections for Lumbosacral Radiculopathy

**DOI:** 10.3390/medicina55050162

**Published:** 2019-05-18

**Authors:** Dong Yoon Park, Seok Kang, Joo Hyun Park

**Affiliations:** 1 Department of Rehabilitation Medicine, Graduate School, The Catholic University of Korea, Seoul 06591, Korea; dyfree@naver.com; 2 Department of Physical Medicine and Rehabilitation, Korea University Guro Hospital, Seoul 08308, Korea; caprock@paran.com; 3 Department of Rehabilitation Medicine, Seoul St. Mary’s Hospital, College of Medicine, The Catholic University of Korea, Seoul 06591, Korea

**Keywords:** radiculopathy, epidural injection, predictor, corticosteroid, electromyography, hyalurodinase

## Abstract

*Background and Objectives:* The purpose of this retrospective study was to identify predictors of short-term outcomes associated with a lumbosacral transforaminal epidural steroid injection (TFESI). *Materials and Methods:* The medical records of 218 patients, who were diagnosed with lumbosacral radiculopathy and treated with a TFESI, were reviewed in this retrospective study. A mixture of corticosteroid, lidocaine, and hyaluronidase was injected during TFESI. Patients with >50% pain relief on the numerical rating scale compared with the initial visit constituted the good responder group. Demographic, clinical, MRI, and electrodiagnostic data were collected to assess the predictive factors for short-term outcomes of the TFESI. *Results:* A multivariate logistic regression analysis demonstrated that a shorter duration of symptoms and a positive sharp wave (PSW)/fibrillation (Fib) observed in electrodiagnostic study (EDx) increased the odds of significant improvement 2–4 weeks after the TFESI. *Conclusions:* Shorter duration of symptoms and PSW/Fib on EDx were predictors of favorable short-term response to TFESI.

## 1. Introduction

Low back pain and radiating pain are common health conditions, with a lifetime prevalence of about 40–60% [1,2,3]. However, no gold standard imaging or laboratory test is available for the diagnosis of lumbosacral radiculopathy. Clinicians arrive at the diagnosis based on a combination of history, physical examination, imaging, and neurophysiological studies. Decisions regarding optimal treatment are also not easy, especially given the various therapeutic approaches available: medications, physiotherapy, exercise, injections, and surgery.

There is controversy regarding the effect of a lumbosacral epidural steroid injection. Some studies have demonstrated the efficacy of epidural steroid injections for the treatment of radiating pain in patients with lumbosacral disk herniation [4,5,6,7]. Other studies have shown that the effectiveness of this procedure is limited [8,9]. Nevertheless, lumbosacral epidural injections have been widely used to treat radicular pain in clinical practice. In addition, this procedure has also been used to treat patients who have undergone previous low back surgery [10].

Three different techniques, including transforaminal, interlaminar, and caudal approaches, have been widely used in clinical practice. The transforaminal epidural steroid injection (TFESI) seems to be better than an interlaminar or a caudal approach for radicular pain [7,11].

Despite the widespread availability of epidural steroid injections, little is known about the factors predicting their success. Studies investigating the predictors of lumbar epidural steroid injections have shown conflicting results [9,12,13,14,15,16,17,18,19,20,21,22,23,24,25,26,27,28]. If the diagnostic information can be used to predict treatment response, it can facilitate the treatment plan. The aim of this study was to determine the factors that represent predictors of short-term outcomes of a lumbosacral TFESI.

## 2. Materials and Methods

### 2.1. Patients

The patients who had undergone lumbosacral TFESI procedures in the Department of Rehabilitation, Korea University Guro Hospital, from March 2014 to October 2017, were included in this study. The electronic medical records of symptomatic patients who were diagnosed with lumbosacral disk herniation, that presented with radiating pain, and were treated with a TFESI, were reviewed. This study was approved by the institutional review board of the relevant institution (Korea university Guro hospital, Approval code: 2017GR0017, date of approval: 16 November 2017). The inclusion criteria were: (i) patients aged over 20 years old; (ii) patients who complained of radiating pain; (iii) definitive diagnosis of lumbosacral disk herniation, which was confirmed by MRI; and (iv) TFESI treatment.

The exclusion criteria were: (i) inadequate data including history, physical examination, and MRI findings; (ii) failure to visit our clinic again 2–4 weeks after the TFESI; (iii) symptoms after traffic accident or workplace injury; (iv) prior lumbosacral epidural steroid injections in the past 3 months; (v) severe motor weakness of the lower extremity (less than a fair grade on a manual muscle test of any the following: hip flexion, knee extension, ankle dorsiflexion, great toe extension, and ankle plantar flexion); (vi) acute bladder or bowel symptoms; (vii) diagnosis of other diseases, such as diabetic polyneuropathy, in addition to lumbosacral radiculopathy, diagnosed either clinically or via electrodiagnostic study (EDx); or (viii) abnormal blood test results. Blood tests were routinely done for all the patients before the epidural injection. Blood tests included a complete blood count, blood chemistry, C-reactive protein, prothrombin time, and activated partial thromboplastin time. Patients with either severe motor weakness (less than a fair grade) or acute bladder and bowel symptoms were referred to a spine surgeon. Based on these criteria, 218 patients were included in this study. Oral analgesia was prescribed to all patients. None of the included patients were involved in other treatment, such as physical therapy.

### 2.2. TFESI

All of the procedures were conducted by two specialists experienced in spinal intervention under fluoroscopic guidance on an outpatient basis. Blood tests, including complete blood count, blood chemistry, erythrocyte sedimentation rate, C-reactive protein, prothrombin time, and activated partial thromboplastin time, were carried out on all the patients before the procedure to determine infection or bleeding risk. Patients taking anti-platelet agents or anticoagulants were asked to discontinue them for a week before the procedure. Informed consent was obtained from each patient. All the procedures were carried out via an extraepineural approach. With the patient lying in a prone position, the C-arm was rotated obliquely to obtain an X-ray image of pedicle and neural foramen. An 89 mm, 23-gauge spinal needle was advanced into the safe triangle. The safe triangle was defined by the pedicle superiorly, the lateral border of the vertebral body laterally, and the outer margin of spinal nerve medially. The contrast agent was spread into the epidural space and externally along the spinal nerve root. A mixture of 20 mg (0.5 cc) of triamcinolone acetonide suspension, 2 cc of 0.5% lidocaine, and 750 IU of hyaluronidase was slowly injected at each level. This mixture has been used to treat acute and chronic low back pain and radicular symptoms via various mechanisms. Corticosteroids exhibit anti-inflammatory effects, as well as interfere with nociceptive C-fiber conduction [29]. Local anesthetics impair peripheral neurotransmission of pain impulses, normalize the hyperalgesic state of the nervous system, and prevent and reduce neuronal plasticity in the central nervous system by reducing the peripheral nociceptive input [30]. Hyaluronidase is a lysing enzyme that disrupts the epidural scar tissue and adhesions. The use of hyaluronidase reduces fibrosis and facilitates the spread of injections [31]. This enzyme is reported to be effective in treating chronic low back pain or radiating pain [32].

All the patients were asked to visit the outpatient clinic 2 weeks after the TFESI to evaluate the short-term therapeutic effect. However, a few patients failed to visit on the scheduled date because of the patient’s personal circumstances. Therefore, we collected the short-term outcome based on the first visit between 2 and 4 weeks after the TFESI.

### 2.3. Review of Clinical Data and Outcomes

A retrospective review of medical records was performed. The following data were reviewed: (i) demographics, (ii) clinical characteristics, (iii) MRI, and (iv) EDx measurements. Demographic data included age and sex. Clinical data included past medical history, symptoms, and physical examination. Past medical history, including the presence of diabetes, hypertension, and previous lumbosacral spine surgery, was reviewed. Any sprain or trauma associated with the low back was assessed just before the onset of symptoms. Duration of symptoms was classified as acute (≤4 weeks), subacute (4–24 weeks), and chronic (≥24 weeks). The nature of pain was evaluated as follows: presence of low back pain, buttock pain, or neurogenic claudication. The straight leg raise test (SLR) and motor and sensory examination were performed. The SLR was considered positive if the radiating symptoms were triggered by lifting the leg less than 60 degrees. The motor exam was considered abnormal if there was weakness associated with any of the following muscle groups on manual muscle testing: hip flexors, knee extensors, ankle dorsiflexors, great toe extensors, and ankle plantar flexors. The sensory exam was considered abnormal if allodynia, hypoesthesia, anesthesia, or paresthesia was detected along the nerve root dermatome.

All the patients underwent lumbar MRI, and all the MRI data were reviewed by a single radiologist specializing in musculoskeletal imaging at our hospital. MRI data included the level of herniated nucleus pulposus (HNP), the presence of nerve root compression or contact, foraminal stenosis, and moderate-to-severe central canal stenosis.

EDx, including a nerve conduction study and needle electromyography, was performed by qualified electromyographers. We used a needle electromyography (EMG) electrode to examine paravertebral and limb muscles, including at least six muscles: gluteus maximus, tensor fascia lata, vastus medialis, tibialis anterior, peroneus longus, and medial gastrocnemius. We reviewed the presence of radiculopathy based on the EDx report. The presence of a positive sharp wave (PSW) or fibrillation (Fib) in the lower limb, and motor unit action potential (MUAP) change, were also reviewed. A MUAP change refers to polyphasia, long duration, and/or large MUAPs in the lower limb muscles.

The short-term response of TFESI was determined by the degree of pain relief at the outpatient clinic 2 to 4 weeks after TFESI. Pain intensity at the initial and follow-up visits was assessed using the numerical rating scale (NRS) (0–10 points, 11 numeric scale). Good responders were defined as the group with a reduction greater than 50% on NRS at the follow-up visit compared with the initial visit. The others were defined as poor responders.

### 2.4. Statistical Analysis

Descriptive statistics were used to report the demographic and clinical data. Categorical variables were described using absolute and relative frequencies. Continuous variables were described using the mean and standard deviation, and discrete variables were described using the median and corresponding ranges. We conducted the independent *t*-test, the Mann–Whitney test, and the chi-squared test to demonstrate the statistical difference of clinical data between the good and poor response groups. The independent *t*-test and Mann–Whitney test were used for continuous variables. The chi-squared test was used for categorical variables.

Univariate logistic regression analysis was first performed for the analysis of the primary outcome. The odds ratios (ORs) and 95% confidence intervals (CIs) were determined to show the reliability of the estimates. Multiple logistic regression analysis was used to identify individual predictors of substantial response suggested by the univariate analysis. All *p* values <0.05 in the univariate analysis were included in the multivariate analysis. Data were analyzed using SPSS software version 20.0 (SPSS Inc., Chicago, IL, USA), and a *p* value <0.05 was considered significant in all analyses.

## 3. Results

Demographic, clinical, and MRI data were collected from all the patients. However, EDx was performed in 150 of the 218 patients. The number of patients presenting with a favorable response 2–4 weeks after TFESI was 133 (61.0%), and the number of poor responders was 85 (39.0%). Demographics, past medical history, spinal surgery history, duration and presentation of symptoms, physical examination, the number of TFESI levels, and MRI and EDx findings were compared between the favorable and poor response groups (Table 1). A statistically significant difference was observed between the two groups regarding history of lumbar surgery, duration of symptoms, neurogenic claudication, the SLR test, PSW/Fib on EDx, and central canal stenosis on MRI. 

The results of univariate and multivariate logistic regression analyses are listed in Table 2. In the univariate regression analysis, history of lumbar surgery, duration of symptoms, neurogenic claudication, the SLR test, PSW/Fib on EDx, and central canal stenosis on MRI findings were associated with good response. The multivariate regression analysis demonstrated that only a shorter duration of symptoms and PSW/Fib findings on EDx were predictors of a favorable response to TFESI. In addition, the absence of past spine surgery and central canal stenosis findings on MRI was also associated with a good response in the multivariate regression analysis, even though the association was not statistically significant.

## 4. Discussion

In this study, we measured short-term outcomes 2–4 weeks after TFESI. Previous studies reported that epidural steroid injections may result in improvement in lumbosacral radicular pain between two and six weeks, which may relate to the duration of the therapeutic effect of corticosteroid [33,34]. All the procedures were extraepineural in this study, because extraepineural injections are less painful and more effective than intraepineural injections [16,33]. The patients from traffic accidents or injuries at industrial sites were excluded, as they were covered by auto-insurance and worker’s compensation in South Korea. The treatment response may be affected by secondary gain [12].

The first finding was that shorter symptom duration was the strongest factor associated with a good response. Having acute symptoms or subacute symptoms increased the odds of significant improvement 2–4 weeks after TFESI compared with manifesting chronic symptoms. These results are consistent with previous studies. Ahilan et al. [28] reported that symptom duration for over a year decreased the odds of improvement 3 months after epidural lumbar steroid injection; however, they did not describe which medication was injected. Copper et al. [13] reported that patients with chronic symptoms tend to display worse outcomes than those with acute symptoms. A study by Cyteval et al. [15] also showed that the symptom duration before periradicular steroid injection for lumbar radiculopathy was highly correlated with pain relief outcomes at two weeks and at the one-year follow-up. Corticosteroid and local anesthetics were used in the above two studies.

A longer duration of symptoms results in chronic inflammation, including fibrosis and necrosis of nerve root fibers [35,36]. These results suggest that corticosteroids are less effective in chronic inflammation. In this study, we added hyaluronidase to steroid and lidocaine for injections, but the outcome was the same as in previous studies. This implies that the previously discussed mechanism of local anesthetics and hyaluronidase, which more likely targets chronic inflammation, is not as effective as the anti-inflammatory effect of corticosteroids.

Various management strategies have been suggested as chronic back pain and radicular pain do not well respond to various approaches. As seen in this study, TFESI is less effective for chronic low back pain. Therefore, we should emphasize non-pharmacologic treatment, such as exercise therapy, especially in chronic low back pain [37].

Whether EDx findings can be a predictor of response to epidural steroid injection has been challenged by several studies. A few studies [20,24,26] reported that radiculopathy in EDx was associated with a favorable outcome to epidural steroid injection, whereas other studies [12,21,22] reported a lack of association. In this study, the diagnosis of lumbosacral radiculopathy by EDx was not a predictive factor of response to TFESI. However, PSW/Fib findings related to limb muscles in patients diagnosed with lumbosacral radiculopathy by EDx increased the odds of improvement with TFESI. When PSW/Fib was detected only in the paravertebral muscle without limb muscles, the findings were not considered as radiculopathy in this study. Similar findings have been detected in 15–42% of asymptomatic individuals, and this increased with the age of the examined population [38,39].

Several explanations are possible. First, the diagnosis of radiculopathy by EDx is limited to motor neuron axonal damage in most cases. It is impossible to diagnose radiculopathy by EDx in the presence of only mild injury at the nerve root, such as focal demyelination without damage to the motor axons. The patients who were diagnosed with radiculopathy but exhibited normal findings on EDx also responded well to TFESI. This hypothesis explains the absence of association between EDx diagnosis and TFESI outcomes. Second, membrane instability characterized by PSW/Fib is generally detected in limb muscles within three weeks after acute neural insult and continues until the muscle fiber is reinnervated or atrophied completely. When the patient was diagnosed with radiculopathy without PSW/Fib, EDx was performed after reinnervation, because completely atrophied muscle would have been excluded from this study due to severe motor weakness. It is reported that the limb muscles are reinnervated about three months after symptom onset. In contrast to PSW/Fib, most of the MUAP changes, including polyphasic, large, and long duration, were found in chronic and static radiculopathy, even though polyphasic MUAP was detected at an early stage [40,41]. Thus, the duration of PSW/Fib correlated with good response to TFESI. Third, the diagnosis of radiculopathy based on MUAP changes alone may involve older and stable lesions, which may not be associated with recent symptoms. The absence of a significant difference between good and poor response groups based on EDx radiculopathy suggests the possibility of diagnosis unrelated to recent symptoms. Fourth, the growth of a bony spur may gradually compress the nerve root, resulting in relatively mild damage to the nerve fibers. In this case, collateral sprouting and denervation may occur at a similar rate with hard-to-observe PSW/Fib initially. The effect of TFESI is limited in this case, because the majority of mechanisms involved mechanical compression by bony spur.

The absence of spine surgery could be associated with favorable response to TFESI, even though the association was only present in the univariate analysis. This result can be explained by postoperative scar formation. Epidural scar from low back surgery probably induced soft tissue adhesion and mechanical compression of the nerve root, which is resistant to the anti-inflammatory effects of corticosteroid. The scar also mechanically prevents delivery into the epidural space and eventually decreases the effectiveness of injection. Previous studies report conflicting results. Sivaganesan et al. [28] reported that back surgery was associated with a poor response to epidural steroid injection, whereas Tong et al. [12] found no significant correlation between them. Neither of these two studies described the type of back surgery included. In this study, any type of back surgery, such as discectomy or posterior lumbar interbody fusion, was included. The degree of scarring is determined by the type of back surgery. Therefore, the association is also influenced by the proportion of various types of back surgeries and requires appropriate classification in future studies.

We were unable to determine the association between MRI findings and a good response to the TFESI in the multivariate regression analysis. However, a central canal stenosis observed on MRI may be associated with increased odds of a positive response, even though the association was not statistically significant in multivariate analysis. These associations have been disputed in previous studies [19,22,23,27,28]. Other variables, including age, sex, the presence of trauma or sprain, the location of pain, neurogenic claudication and findings of physical examination, cannot be considered as predictors of short-term outcome after a TFESI. 

In this study, the short-term success rate of a TFESI at 2–4 weeks was 61%. Previous studies have reported that the success rate is about 34–78% and the success rate of the short-term effect is about 50%, which is similar to our study [8,42]. One systemic review [42] reported that the level of evidence in support of epidural steroid injections is moderate. However, many studies [9,43] have shown controversial results about the long-term effects of the procedure.

This study has several limitations. First, the outcome was only evaluated by a change in NRS. There are specific tools designed for evaluation of function and pain in patients with low back pain, such as the Oswestry Disability Index and the Pain Disability Questionnaire. Because it is difficult to objectively assess response to treatment, these tools can be also used simultaneously to assess pain, function, or emotion in a variety of ways. This is a limitation of retrospective studies analyzing data derived from pre-existing medical records. Second, the predictive factors suggested in this study are limited to short-term response to TFESIs. It was impossible to accurately and reliably predict long-term treatment response to lumbar TFESIs based on short-term pain relief [44]. Additional studies investigating long-term response are needed. Third, the location of the TFESI was not standardized. In our clinic, the most frequently targeted levels of TFESI were L4/L5, L5/S1, and S1 foramens, while L2/3 or L3/4 were rarely targeted. About two levels of TFESI were administered to both good and poor responders. However, the details of the injection level were not described in this study. Fourth, the difference between good and poor responders could be attributable to the natural course of the condition in the patients, such as chronicity of pain. Future studies comparing the natural course between two groups will be needed. Fifth, the patients were re-assessed in a 2–4 week range after the injection. Although 2–4 weeks is a short period of time compared to other studies [42], the pain response at two weeks after injection can be different to the response at four weeks.

## 5. Conclusions

The short duration of symptoms and the presence of PSW/Fib findings in lower limb muscles in EDx diagnosis are likely to predict a favorable response 2–4 weeks after a TFESI in patients diagnosed with lumbar disk herniation on MRI and who present with radiating leg pain. Clinicians should consider these factors in the risk–benefit analysis of TFESIs. The results should be validated via randomized prospective studies in the future.

## Figures and Tables

**Table 1:** Summary of Results.

Variables		Values		
		Poor Responders (*n* = 85)	Good Responders (*n* = 133)	*p*-Value
Pain at initial visit (NRS)		7.51 ± 1.297	7.91 ± 1.048	0.003
Demographic				
Sex	Male	38 (44.7%)	58 (43.6%)	0.874
	Female	47 (55.3%)	75 (56.4%)	
Age		60.31 ± 16.35	56.31 ± 14.44	0.060
Clinical				
Diabetes	Yes	9 (10.6%)	11 (8.3%)	0.563
	No	76 (89.4%)	122 (91.7%)	
Hypertension	Yes	26 (30.6%)	33 (24.8%)	0.349
	No	59 (69.4%)	100 (75.2%)	
Previous spine surgery	Yes	13 (15.3%)	6 (4.5%)	0.006
	No	72 (84.7%)	127 (95.5%)	
Trauma or sprain	Yes	6 (7.1%)	19 (14.3%)	0.102
	No	79 (92.9%)	114 (85.7%)	
Duration of symptoms	weeks	60.75 ± 79.14	16.53 ± 48.01	0.000
	Chronic	45 (52.9%)	17 (12.8%)	0.000
	Subacute	15 (17.6%)	30 (22.6%)	
	Acute	25 (29.4%)	86 (64.7%)	
Low back or buttock pain	Yes	50 (58.8%)	92 (69.2%)	0.118
	No	35 (41.2%)	41 (30.8%)	
Neurogenic claudication	Yes	19 (22.4%)	15 (11.3%)	0.028
	No	66 (77.6%)	118 (88.7%)	
SLR test	Full	73 (86.9%)	94 (74.0%)	0.024
	Positive	11 (13.1%)	33 (26.0%)	
Motor exam	Normal	77 (90.6%)	109 (82.0%)	0.079
	Abnormal	8 (9.4%)	24 (18.0%)	
Sensory exam	Normal	72 (84.7%)	109 (82.0%)	0.598
	Abnormal	13 (15.3%)	24 (18.0%)	
MRI				
No. of HNP levels	1	14 (18.7%)	35 (28.7%)	0.160
	2	22 (29.3%)	39 (32.0%)	
	>2	39 (52.0%)	48 (39.3%)	
Root compression or contact	Yes	58 (77.3%)	101 (82.8%)	0.346
	No	17 (22.7%)	21 (17.2%)	
Foraminal stenosis	Yes	43 (57.3%)	79 (64.8%)	0.298
	No	32 (42.7%)	43 (35.2%)	
Central canal stenosis	Yes	34 (45.3%)	33 (27.0%)	0.009
	No	41 (54.7%)	89 (73.0%)	
EDx				
EDx diagnosis	Normal	22 (35.5%)	26 (29.5%)	0.443
	Radiculopathy	40 (64.5%)	62 (70.5%)	
PSW/Fib	Yes No	10 (16.1%) 52 (83.9%)	32 (36.4%) 56 (63.6%)	0.007
MUAP change	Yes	37 (59.7%)	50 (56.8%)	0.727
	No	25 (40.3%)	38 (43.2%)	
No. of levels of TFESI		2.15 ± 0.60	2.04 ± 0.41	0.093

Data are expressed as mean ± standard deviation or absolute number (percentage). Abbreviations: EDx, electrodiagnosis; HNP, herniated nucleus pulposus; NRS, numerical rating scale; TFESI, transforaminal epidural steroid injection; PSW, positive sharp wave; Fib, fibrillation; MUAP, motor unit action potential.

**Table 2:** Univariate and multivariate analysis of variables.

		Univariate		Multivariate	
		OR (95%CI)	*p*-Value	OR (95%CI)	*p*-Value
Demographic					
Sex	Male	1			
	Female	0.956 (0.553–1.654)	0.874		
Age		0.982 (0.964–1.001)	0.061		
Clinical					
Diabetes	Yes	1			
	No	1.313 (0.520–3.317)	0.564		
Hypertension	Yes	1			
	No	1.335 (0.728–2.449)	0.350		
Previous spine surgery	Yes	1			
	No	3.822 (1.392–10.489)	0.009	3.496 (0.568–21.528)	0.177
Trauma or sprain	Yes	1			
	No	0.456 (0.174–1.192)	0.109		
Duration of symptoms	Chronic	1		1	
	Subacute	5.294 (2.299–12.189)	0.000	6.772 (2.034–22.546)	0.002
	Acute	9.106 (4.459–18.594)	0.000	7.080 (2.582–19.413)	0.000
Low back or buttock pain	Yes	1			
	No	0.637 (0.361–1.123)	0.119		
Neurogenic claudication	Yes	1			
	No	2.265 (1.079–4.751)	0.031	1.462 (0.489–4.289)	0.489
SLR test	Full	1			
	Positive	2.330 (1.103–4.921)	0.027	1.358 (0.413–4.464)	0.614
Motor exam	Normal	1			
	Abnormal	2.119 (0.904–4.967)	0.084		
Sensory exam	Normal	1			
	Abnormal	1.219 (0.583–2.550)	0.598		
MRI					
No. of HNP levels	1	1			
	2	0.709 (0.315–1.595)	0.406		
	>2	0.492 (0.233–1.042)	0.064		
Root compression or contact.	Yes	1			
	No	0.709 (0.347–1.452)	0.347		
Foraminal stenosis	Yes	1			
	No	0.731 (0.406–1.139)	0.298		
Central canalStenosis	Yes	1			
	No	2.237 (1.221–4.096)	0.009	2.291 (0.908–5.780)	0.079
EDx					
EDx diagnosis	Normal	1			
	Radiculopathy	1.312 (0.656–2.623)	0.443		
PSW/Fib	No	1			
	Yes	2.971 (1.330–6.641)	0.008	3.889 (1.359–11.133)	0.011
MUAP change	Yes	1			
	No	0.889 (0.460–1.720)	0.727		

Abbreviations: EDx, electrodiagnosis; Fib, fibrillation; HNP, herniated nucleus pulposus; MUAP, motor unit action potential; NRS, numerical rating scale; PSW, positive sharp wave; TFESI, transforaminal epidural steroid injection.
